# Change of intestinal microbiota in cerebral ischemic stroke patients

**DOI:** 10.1186/s12866-019-1552-1

**Published:** 2019-08-19

**Authors:** Na Li, Xingcui Wang, Congcong Sun, Xinwei Wu, Mei Lu, Youfeng Si, Xiang Ye, Tan Wang, Xiaolin Yu, Xinjing Zhao, Na Wei, Xingbang Wang

**Affiliations:** 1 Department of Dermatology, Affiliated Hospital of Shandong University of Traditional Chinese Medicine, Jinan, 250011 Shandong China; 2 Department of Nephrology, Qilu Children’s Hospital of Shandong University, Jinan, 250022 China; 3 Department of Neurology, Qilu Hospital of Shandong University, Jinan, 250012 China; 4 Department of Geriatric Medicine, Qilu Hospital of Shandong University; Key Laboratory of Cardiovascular Proteomics of Shandong Province, Qilu Hospital of Shandong University, Jinan, 250012 China; 5 Department of Neurology, Feicheng Mining Center Hospital of Shandong Province, Feicheng, Tai an, 271608 China

**Keywords:** Cerebral ischemic stroke, Microbiota, Dysbiosis, Outcomes

## Abstract

**Background:**

Gut microbiota has been suggested to play a role in stroke patients. Nevertheless, little is known about gut microbiota and the clinical indexes in stroke patients.

**Methods:**

Total of 30 cerebral ischemic stroke (CI) patients and 30 healthy control were enrolled in this study and the fecal gut microbiota was profiled via Illumina sequencing of the 16S rRNA V1-V2. The National Institutes of Health Stroke Scale (NIHSS) were used to quantify stroke severity and modified Rankin scale (mRS) to assess outcome for CI patients. The correlations between the clinical indexes and microbiota were evaluated.

**Results:**

Though the microbial α-diversity and structure is similar between CI patients and healthy controls, the gut microbiota of CI patients had more short chain fatty acids producer including *Odoribacter*, *Akkermansia, Ruminococcaceae_*UCG_005 and *Victivallis*. We also found that the special microbes were correlation with serum index, such as norank_O*_ _Mollicutes_RF9*, *Enterobacter*, *Ruminococcaceae*_UCG-002 were negative correlation with LDL (r = − 0.401, *P* < 0.01), HDL (r = − 0.425, *P* < 0.01) and blood glucose (r = − 0.439, *P* < 0.001), while the HDL was significantly positive correlation with the genus *Ruminococcus*_1 (r = 0.443, *P* < 0.001). The *Christensenellaceae*_R-7_group and norank_f_*Ruminococcaceae* was significantly positive correlation with NIHSS1M (r = 0.514, *P* < 0.05; r = 0.449, *P* < 0.05) and mRS (r = 0.471, *P* < 0.05, r = 0.503, *P* < 0.01), respectively. On the other hand, the genus *Enterobacter* was significantly negative correlation with NIHSS1M (r = 0.449, *P* < 0.05) and mRS (r = 0.503, *P* < 0.01).

**Conclusions:**

This study suggests that CI patients showed significant dysbiosis of the gut microbiota with enriched short chain fatty acids producer, including *Odoribacter*, *Akkermansia*. This dysbiosis was correlation with the outcomes and deserves further study.

**Electronic supplementary material:**

The online version of this article (10.1186/s12866-019-1552-1) contains supplementary material, which is available to authorized users.

## Background

Cerebrovascular accidents (stroke) is brain injury caused by disruption the supply of blood to a brain region, which could result in permanent neurological deficits or death [1]. Now, stroke is global health problem and now became to the second leading cause of death as well as the third leading cause of disability [2]. Basing on the underlying pathology, the stroke was classified to ischemic and hemorrhagic stroke [2]. The cerebral Ischemic stroke (CI) is responsible for 85% of all strokes, and the hemorrhagic stroke accounts for about the rest 15% [2]. The most ischemic stroke is caused by the middle cerebral artery occlusion, resulting in the brain tissue damage in the affected territory, which is followed by inflammatory and immune response [1]. Despite the stroke debilitated the neurological deficits, infection is the superior cause of death after stroke [3]. It was found that 90 % of stroke cases may be correlated with behavioral factors including poor diet, smoking, and low physical activity as well as metabolic factors including obesity, hypertension, diabetes mellitus. Previous study also found that gut microbiota might be as a risk factor for stroke [4].

The gastrointestinal tract is thought to be a major immune organ which was equipped with the largest pool of immune cells, accounting for more than 70% of the entire immune system. More and more evidences propose that the gut inflammatory along with immune response plays an essential role in the pathophysiology of stroke and this may become an important therapeutic target for treatment of stroke [5]. It was found that gut microbiota plays a vital role in regulation of the immune system. The gut microbiota was also revealed to be an important influence factor for stroke in a mouse model [6–9]. In addition, stroke usually cause gut dysmotility, gut microbiota dysbiosis, gut hemorrhage, as well as gut-origin sepsis, resulting in poor prognosis. However, there are little about the characteristics of gut microbiota from stroke patient [10].

This study aims to determine the characteristic of gut microbiota of stroke patient. We also evaluated the relations between gut microbiota and serum indexes or outcome by Spearman’s rank correlation.

## Results

### Subjects

Total of 60 subjects, including 30 CI (21 males, 9 females) and 30 healthy control (18 males, 12 females) were recruited in this study (Table 1). It was found that there were no significant differences in gender (male: 70. % vs. 60.0%, *P* = 0.42) and age (60.47 ± 10.57 vs. 64.17 ± 12.67, *P* = 0.31) between the CI group and healthy control group. The LDL, GLU, UA, TG, HCY of CI patients were similar with the healthy control. The HDL of CI patients was lower than that of healthy control group (1.10 ± 0.19 vs. 1.29 ± 0.24, *P* < 0.01) (Table 1). The NIHSS at 7d (NIHSS 1 W) and 1 month (NIHSS 1 M), modulate RANK score (mRS) and serum index of each subjects were shown in Additional file 1: Table S1 and Additional file 2: Table S2.

**Table 1:** Characteristics of the study participants

	CI (*n* = 30)	HC (n = 30)	*P*
Sex (male, %)	21 (70%)	18 (60%)	0.42
Age	60.47 ± 10.57	64.17 ± 12.67	0.31
LDL	2.20 ± 0.68	2.30 ± 0.50	0.51
HDL	1.10 ± 0.19	1.29 ± 0.24	< 0.01
UA	303.8 ± 64.5	282.3 ± 62.7	0.20
GLU	4.92 ± 0.67	5.20 ± 0.76	0.14
HCY	12.94 ± 3.93	12.36 ± 3.33	0.54
TG	1.24 ± 0.47	1.33 ± 0.31	0.39

*HDL* high-density lipoprotein, *LDL* low-density lipoprotein, *GLU* glucose of blood, *UA* uric acid, *TG* triglycerides, *HCY* homocysteine

### The microbial α-diversity and structure is similar between CI and HC group

We obtained a total of 2,191,980 high-quality sequences by quality-filtering with a coverage above 99.0%. After removing rare OTUs., 19,869 sequences per sample were obtained which were clustered to 955 OTUs. The results of bacterial community richness and diversity were shown in Table 2. It was found that there was no difference in shannon, simpson, ace or chao index between two group.

**Table 2:** The index of α-diversity

	CI (*n* = 30)	HC (*n* = 30)	*P*
Shannon	3.69 ± 0.52	3.59 ± 0.59	0.3515
Simpson	0.09 ± 0.06	0.10 ± 0.07	0.4786
Ace	555.68 ± 51.24	552.19 ± 43.47	0.4679
Chao	565.64 ± 55.61	558.79 ± 48.05	0.7775

**Fig. 1:**
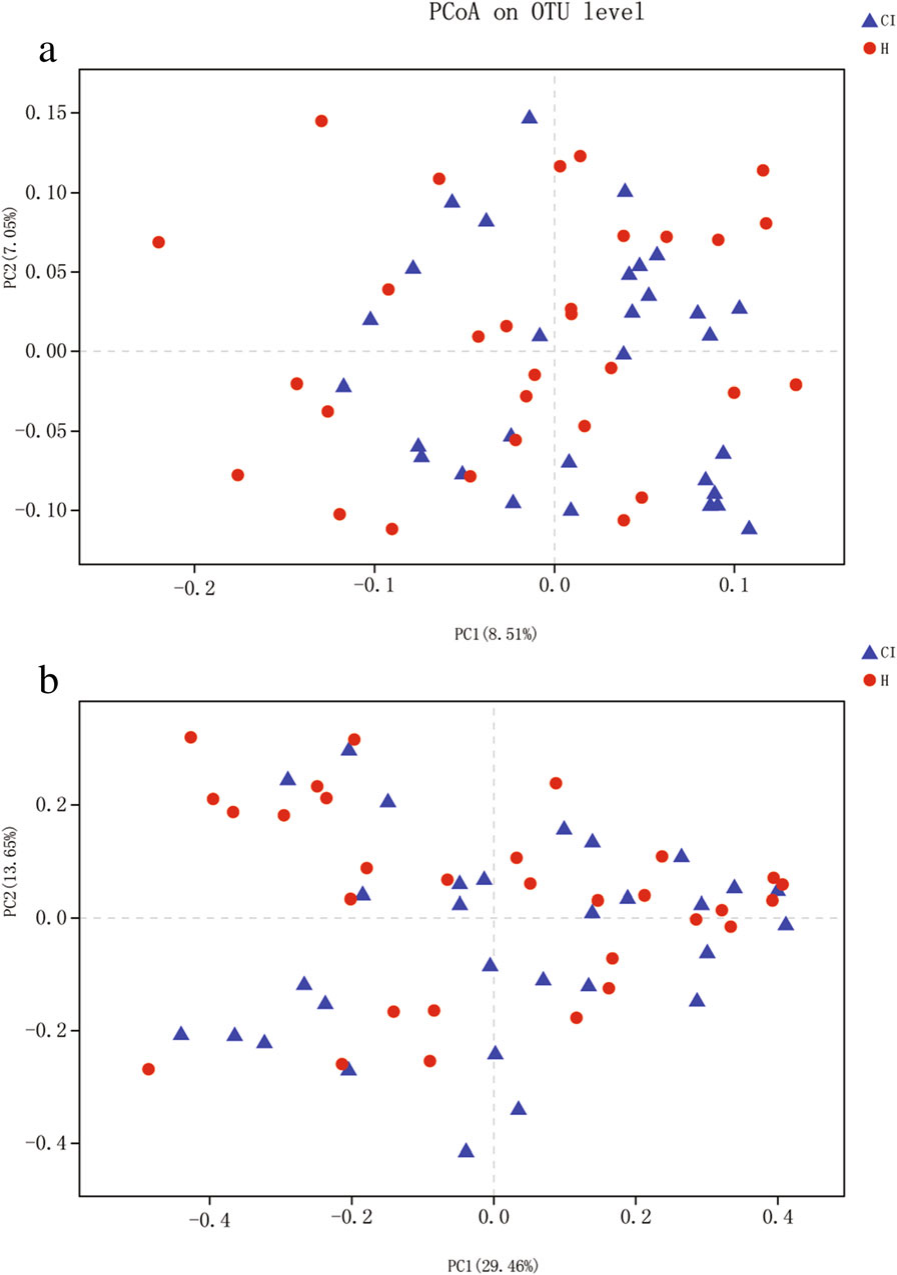
PCoA analysis of the microbiota among CI and healthy control groups. **a** Weighted unifrac PCoA; **b** Unweighted unifrac PCoA

The similarity of the bacterial community structures between CI group and healthy control group was evaluated by PCoA (Fig. 1). There was no obvious difference of the microbiota structure between the 2 groups. Then, ANOSIM were performed and also found a high similarity in the bacterial community between CI patients and the healthy control based on two algorithms (unweighted unifrac, r = 0.0254, *P* = 0.132; weighted unifrac r = − 0.012, *P* = 0.649, respectively).

### Differentially abundant taxon in microbiota of CI patients and healthy control

**Fig. 2:**
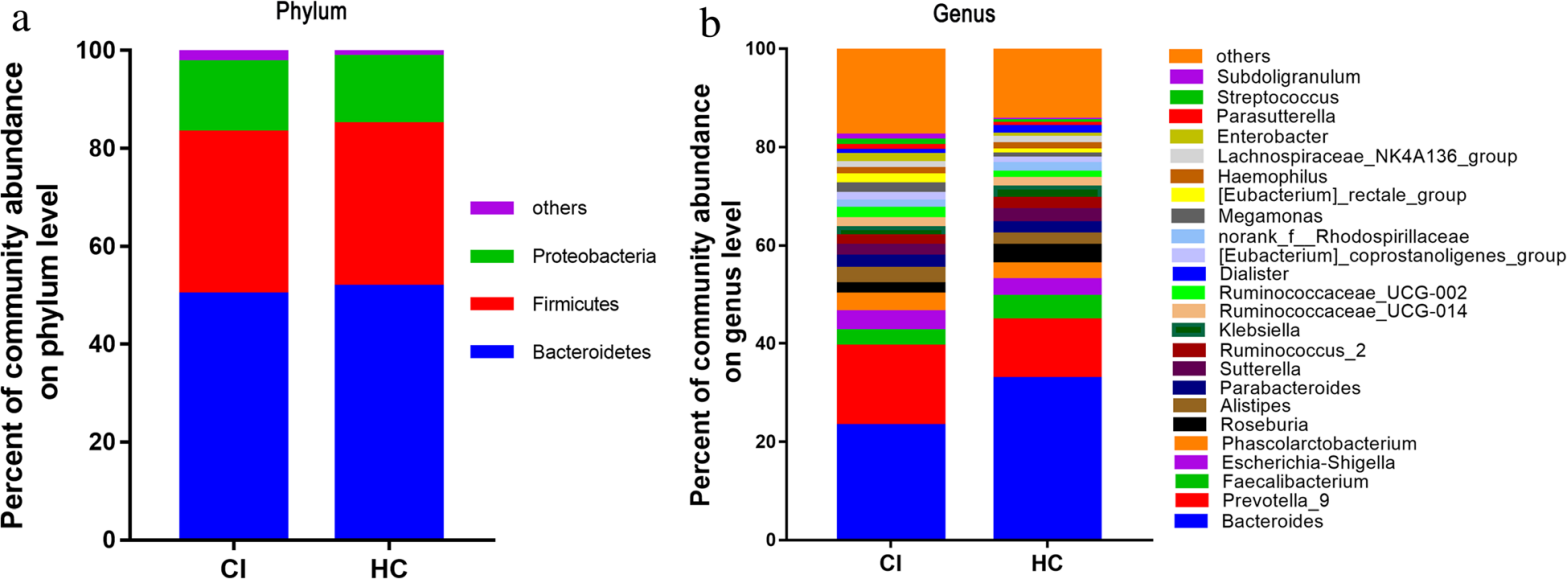
The relative taxa abundance between CI and healthy control groups. **a** Phylum; **b** Genus

**Fig. 3:**
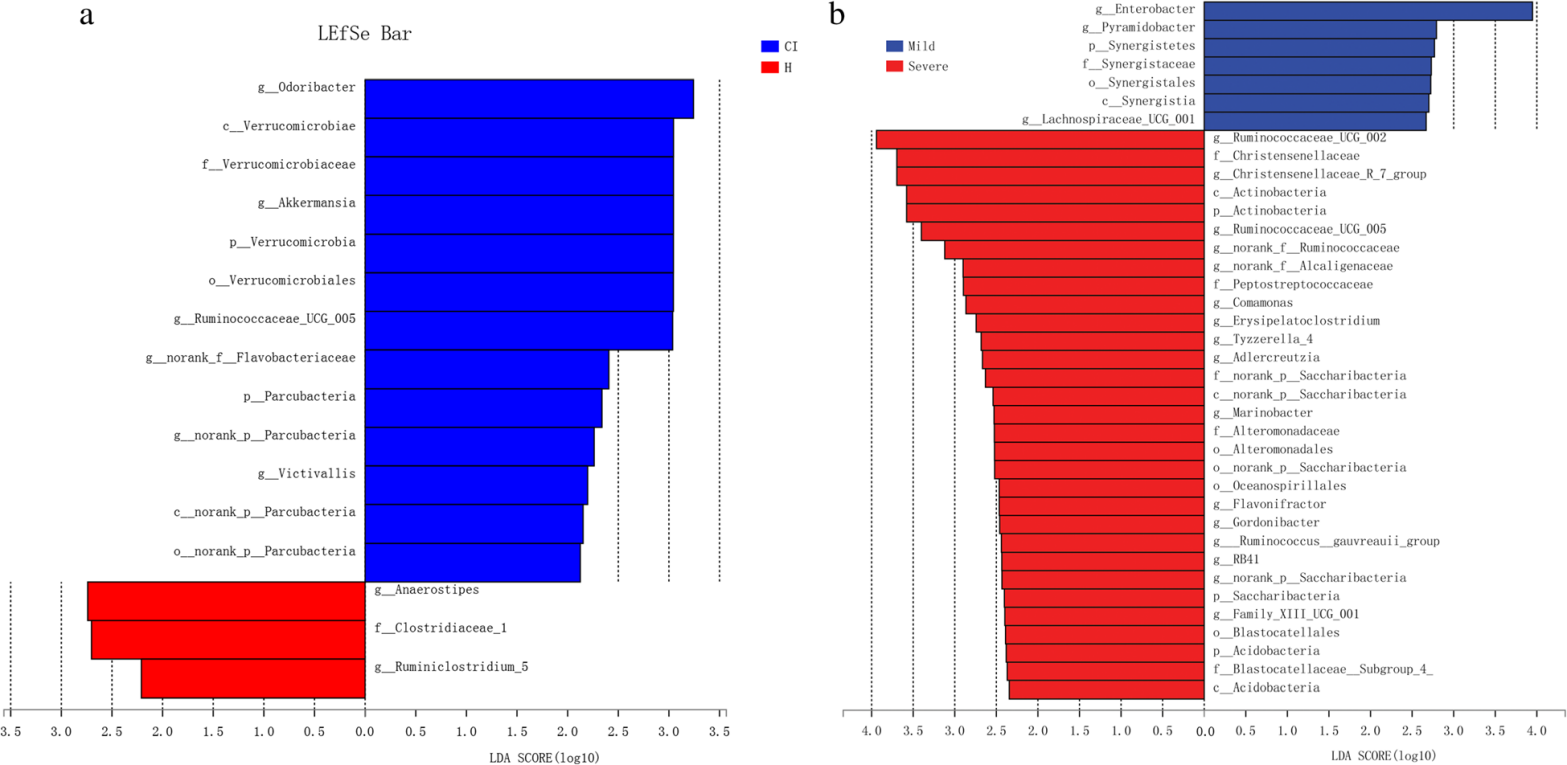
**a** The most differentially abundant taxa between CI and **b** healthy control group based on LEfSe analysis

Most of the gut bacteria detected in this study falls into 3 phyla: Bacteroidetes, Firmicutes, and Proteobacteria (Fig. 2a). The main genus of gut micriobiota (the percentages were above 1%) includes 24 genera and they compose up to 80% of the total microbiota, such as *Bacteroides*, *Prevotella*, *Faecalibacterium*, *Escherichia/Shigella*, *Phascolarctobacterium*, and *Roseburia* (Fig. 2b). The taxa that most likely reveal the differences between CI and healthy control group were identified by LEfSe (Fig. 3). At the genus level, *Anaerostipes* and *Ruminiclostridium_*5 were significantly enriched in the healthy group, while *Odoribacter*, *Akkermansia*, *Ruminococcaceae_*UCG_005, *norank_p_Flavobacteriaceae*, *norank_p_Parcubacteria*, and *Victivallis* exhibited relatively higher abundance in the CI group (Fig. 3a). We also observed that the genus *Enterobacter*, *Pyramidobacter*, and *Lachnospiraceae*_UCG_001 increased in mild CI patients (NIHSS score ≤ 4) (Fig. 3b). While, genus Ruminococcaceae_UCG-002, Christensenellaceae_R-7_group, Ruminococcaceae_UCG-005 and norank_f_Ruminococcaceae increased in severe stroke patients (NIHSS score > 4) (Fig. 3b).

### The gut microbiota was related to the clinical indices

**Fig. 4:**
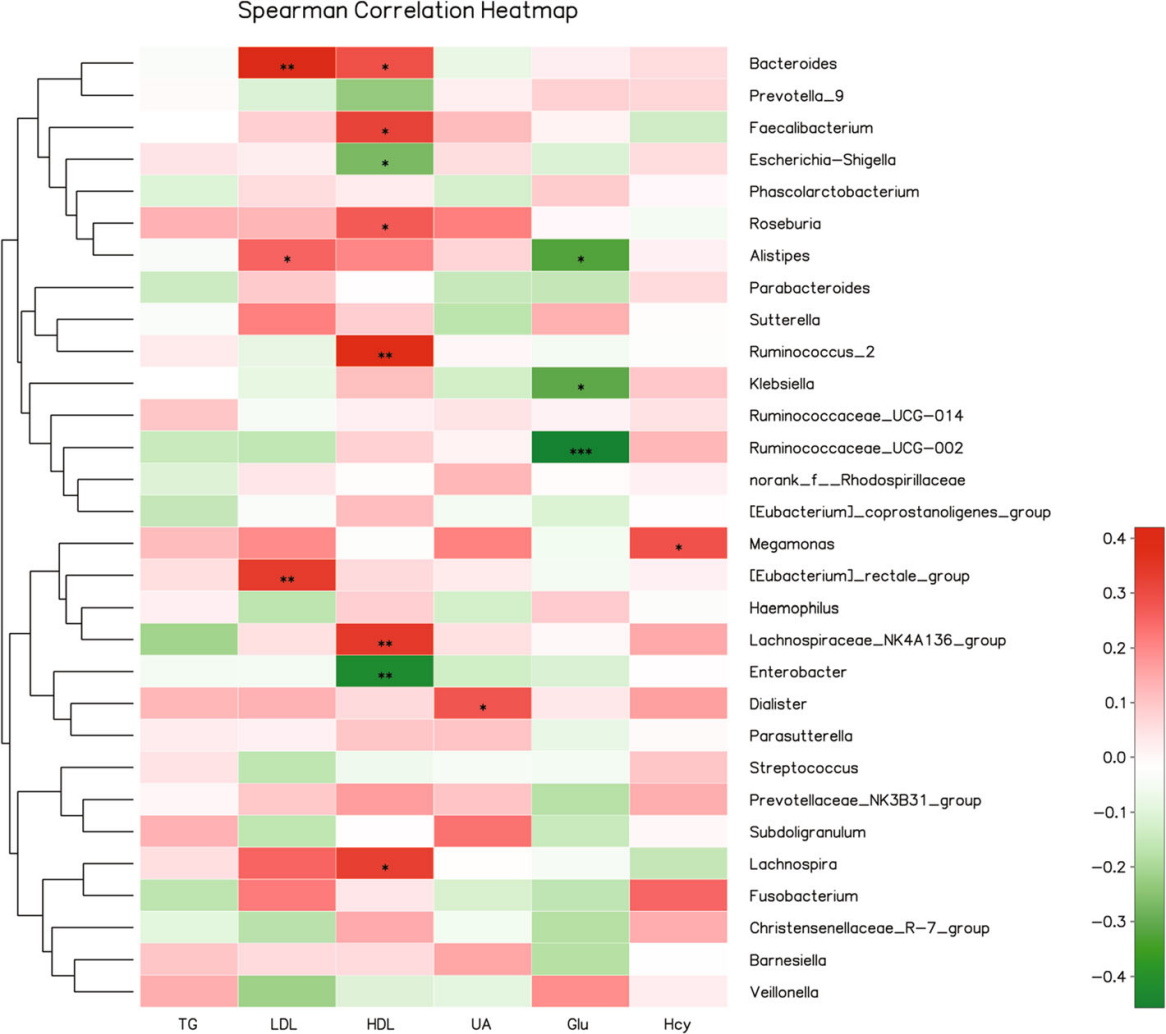
Heatmap of spearman correlation analysis between the gut microbiota and the serum indices. *p < 0.05, **p < 0.01, ***p < 0.01. HDL, high-density lipoprotein; LDL, low-density lipoprotein; GLU, glucose of blood; UA, uric acid; TG, triglycerides; HCY, homocysteine

The serum indices of each subjects were shown in Additional file 1: Table S1. We try to find relationship between the gut microbiota and the serum indices including TG, LDL, HDL, UA, Glu, and Hcy by Spearman’s rank correlation coefficient. We found strong correlations (r > 0.22 or < − 0.22, *P* < 0.05) between them as shown in Fig. 4. A significant positive correlation was exhibited between LDL and genus *Bacteroides* (r = 0.42, *P* < 0.01) as well as [Eubocterium]_rectole_group (r = 0.336, *P* < 0.01). While, it was found that norank_O*_ _Mollicutes*_RF9 *was negative* correlation with LDL (r = − 0.401, *P* < 0.01). The HDL was significantly positive correlation with the genus *Ruminococcus*_1 (r = 0.443, *P* < 0.001), Ruminococcus_2 (r = 0.381, *P* < 0.01) and *Lachnospiraceae*_NK4A136_group (r = 0.338, *P* < 0.01) and negative correlation with the genus *Enterobacter* (r = − 0.425, *P* < 0.01). The UA was found positive correlation with *Dialister* (r = 0.279, *P* < 0.05) and the GLU was found significantly negative correlation with *Ruminococcaceae*_UCG-002 (r = − 0.439, *P* < 0.001), *Alistipes* (r = − 0.313, *P* < 0.05), and *Ruminococcus*_1 (r = − 0.287, *P* < 0.05). And the Hcy was found positive correlation with *Megamonas* (r = 0.291, *P* < 0.05) and *Fusobacterium* (r = 0.297, *P* < 0.05).

### The gut microbiota was correlated with the severity and outcome of CI

**Fig. 5:**
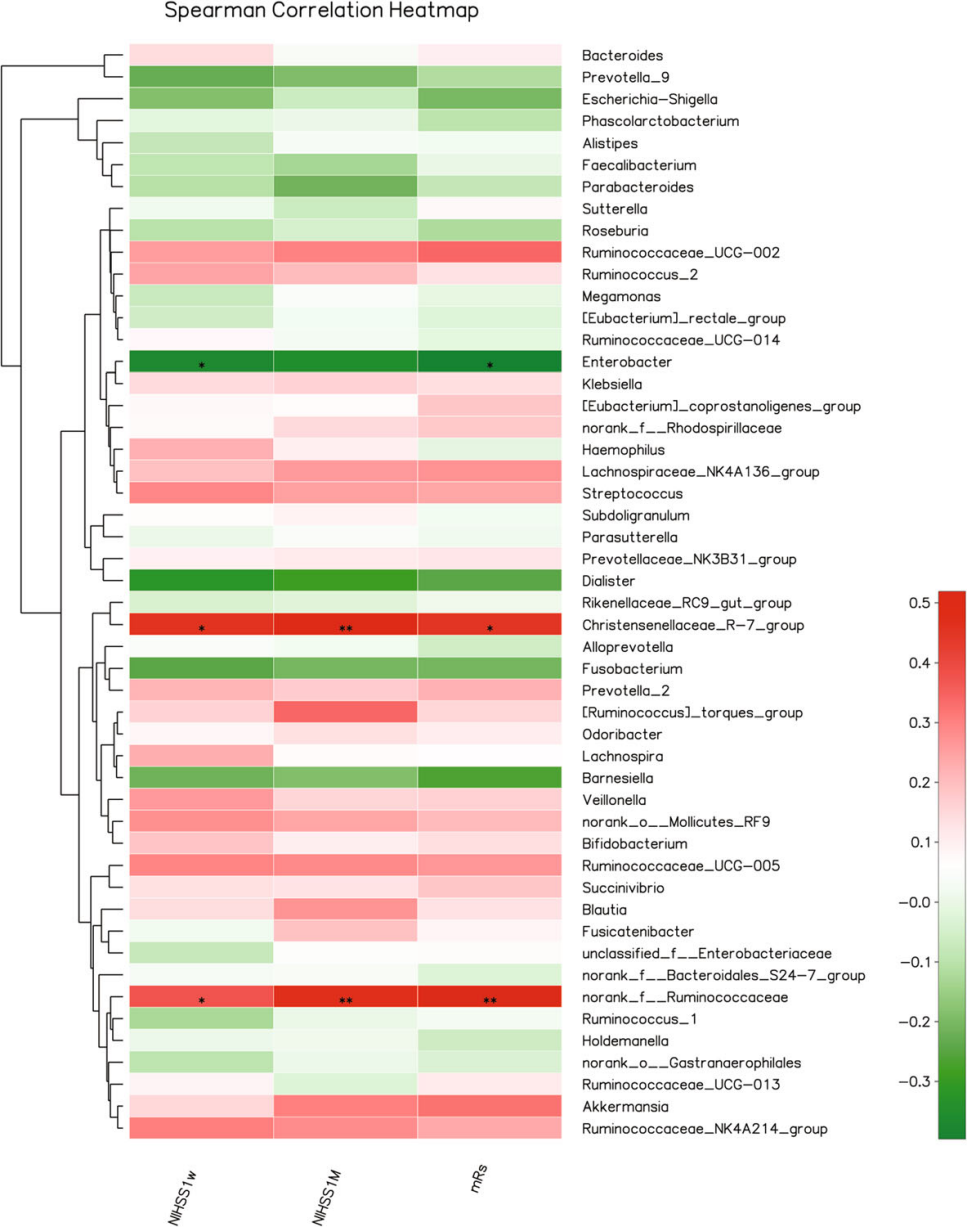
Heatmap of spearman correlation analysis between the gut microbiota and the outcome of CI patients. *p < 0.05, **p < 0.01, ***p < 0.01

The severity and outcome of CI patients could be evaluated by NIHSS and mRS (Additional file 2: Table S2). Then, we do the Spearman’s rank correlation coefficient analysis and attempt to explore the relationship between the gut microbiota and severity or outcome including NIHSS 1 W NIHSS 1 M and mRS (Fig. 5). As a result, the genus *Christensenellaceae*_R-7_group was significantly positive correlation with NIHSS1W (r = 0.488, *P* < 0.01), NIHSS1M (r = 0.514, *P* < 0.05) and mRS (r = 0.471, *P* < 0.05). While, the genus norank_f_*Ruminococcaceae* was also significantly positive correlation with NIHSS1M (*r* = 0.449, *P* < 0.05) and mRS (*r* = 0.503, *P* < 0.01). In contrast, the genus *Enterobacter* was significantly positive correlation with NIHSS1M (*r* = 0.449, *P* < 0.05) and mRS (*r* = 0.503, *P* < 0.01).

## Discussion

In this study, the results demonstrate that certain changes in fecal microbiota was happened in ischemic stroke patients, such as the increasement short-chain fatty acids (SCFAs)-producing taxonomies *Odoribacter* and *Akkermansia*. Furthermore, the levels the genus *Christensenellaceae*_R-7_group, norank_f_*Ruminococcaceae*, and *Enterobacter* were correlated with the severity, while the level of genus *Christensenellaceae*_R-7_group was positively correlated with the outcome of CI patients. In addition, we also found that the microbiota was correlated with the human hematological index.

Previous studies have proved that gut microbiota play a vital role in stroke. It was reported that depletion of gut bacteria via antibiotic administration decreased the survival rate of mice following the induction of ischemia [11]. In mice, Houlden et al., found that the composition of caecal microbiota was altered by experimental stroke, such as specific changes of *Peptococcaceae* and *Prevotellaceace* [6]. Benakis et al. found that the microbiota dysbiosis would affect the ischemic stroke outcome via suppressing the traffick of effector T cells from the gut to the leptomeninges in middle cerebral artery occlusion model [9]. Though only a few studies have investigated the role of microbiota in stroke patient, dysregulation of the microbiota in patients following stroke have been identified [12–14]. Yin et al., found that ischemic stroke was associated with different microbiota structure and more opportunistic pathogens, such as *Megasphaera*, *Enterobacter*, *Oscillibacter*, along with fewer commensal or beneficial genera such as *Prevotella*, *Bacteroides*, and *Faecalibacterium* [13]. After that, Yamashiro et al., found that ischemic stroke was independently associated with increasement of Atopobium cluster and *Lactobacillus ruminis*, and decreased numbers of *L. sakei* subgroup based on qRT-PCR [12]. In another study, gut microbiota dysbiosis was identified with increased abundance of *Escherichia*, *Bacteroides*, *Megamonas*, *Parabacteroides*, and *Ruminococcus* in the CI and IS patients compared with the healthy group. In the higher risk of stroke people, there was no significant change in alpha diversity index of the microbiota. However, enrichment of opportunistic pathogens together with low abundance of butyrate-producing bacteria were found in subjects with high risk of stroke [4]. In this study, we also found that the microbial α-diversity and structure was similar between CI patients and healthy control. However, the *Anaerostipes* and *Ruminiclostridium_*5 were significantly enriched in the healthy group, while *Odoribacter*, *Akkermansia*, *Ruminococcaceae_*UCG_005, *norank_p_Flavobacteriaceae*, and *Victivallis* represented a relatively higher abundance in CI group (Fig. 2a). Of them, *Odoribacter*, *Akkermansia, Ruminococcaceae_*UCG_005 and *Victivallis* are known producer of SCFAs, including acetate, propionate, and butyrate [4, 15–17]. Meanwhile, significant differences within the CI group was also observed that the *Enterobacter*, *Pyramidobacter*, and *Lachnospiraceae*_UCG_001 increased in mild CI patients (NIHSS score ≤ 4). While, genus *Ruminococcaceae*_UCG-002, *Christensenellaceae*_R-7_group, *Ruminococcaceae*_UCG-005 and norank_f_*Ruminococcaceae* increased in severe stroke patients (NIHSS score > 4) (Fig. 2b). These results suggest that the intestinal flora may have been involved in the human stroke and correlated to the severity of CI.

SCFAs are a major class of bacterial metabolites obtained from fermentation of otherwise indigestible polysaccharides (fibers) in the colon by bacteria [18]. Preview studies indicated that SCFAs have important role in maintaining the intestinal integrity and anti-inflammatory. As a result, beneficial effects of SCFAs have been found in several disease models, such as colitis, metabolic syndrome and so on [19]. In this study, we found that short-chain fatty acids (SCFAs)-producing taxonomies *Odoribacter* and *Akkermansia* increased in CI patient. *Akkermansia muciniphila* is the single species of genus *Akkermansia* in the human which accounts 1–3% of the total bacterial cells in feces [20]. It was found that *A. muciniphila* was inversely associated with diabetes, obesity, cardiometabolic diseases and low-grade inflammation [16]. Previous study demonstrated that the shift in mucosal microbial composition induced by the stroke were an increasement of *A. muciniphila* along with an excessive abundance of Clostridial species in mice [8]. However, it was reported that *Akkermansia* decreased in CI patient [14]. The *Akkermansia* was of 20% abundance of the total microbe in one normal control and this might cause statistical bias. In this study, we found that *Akkermansia* significantly increased in CI patient (*P* < 0.05) (Fig. 2c). It was reported that *A. muciniphila* could use mucin to produce high levels acetate which may be consumed by butyrate-producing Ruminococcaceae to promote butyrate production [4]. In this study, genus *Odoribacter*, a butyrate producer which belongs to phylum Bacteroidetes [21], also increase in CI patient. This indicated that the simultaneous increase of these two bacteria might promote the production of butyrate. Butyrate is a preferential energy source for epithelial cells and it preserves the epithelial heath [22]. Butyrate also could impact the expression level of genes which are aroused by *A. muciniphila* in epithelial cells [23]. Therefore, Stanley et al. thought that the *Akkermansia* may play a crucial role in healing of wound damage via promotion the butyrate levels, resulting in consolidate epithelial integrity in the post-stroke mice [8]. In addition, *A. muciniphila* could induce mucus production and expression of Reg3γ in colon, resulting microbiota remodeling [24]. Thus, further work needs to be done to find out the possible role of *Akkermansia* and *Odoribacter* in post-stroke.

Regarding the sequelae of a stroke, predicting the severity and outcome is important to CI patients and their families in guiding the treatment [25]. Many biomarkers offer the potential to predict the outcome of stroke, including TG/HDL-C [26], IL-6, NT-proBNP [27], and serum YKL-40 [28]. In this study, the genus *Christensenellaceae*_R-7_group and norank_f_*Ruminococcaceae* was significantly positive correlation with NIHSS1M (r = 0.514, *P* < 0.05; r = 0.449, *P* < 0.05) and mRS (r = 0.471, *P* < 0.05, r = 0.503, *P* < 0.01), respectively. On the other hand, the genus *Enterobacter* was significantly negative correlation with NIHSS1M (r = 0.449, *P* < 0.05) and mRS (r = 0.503, *P* < 0.01). Yin et al. found that opportunistic pathogens including *Enterobacter* enriched in stroke patients [13]. However, the relationship between genus *Enterobacter* and the severity or outcome is unknown. We also found that the genus *Enterobacter* was negative correlation with the HDL-C (r = − 0.425, *P* < 0.01), which was considered as a potential protective factor against CVD [29]. Thus, these results suggested that the HDL-C was positive correlation with the NIHSS at 1 month (r = 0.449, *P* < 0.05) and mRS and this is inconsistent with previous studies [30]. On the other hand, we also found that the genus *Enterobacter* increased in mild CI patients (NIHSS score ≤ 4). These patients usually have a better outcome than severe CI patients. These might be the reason that the genus *Enterobacter* was negative correlation with NIHSS 1 M and mRS. Until now, we knew little about the genus of Christensenellaceae_R-7_group and norank_f_Ruminococcaceae. Further study to identify the potent microbial biomarkers for predicting the outcome of stroke patients need to be done.

## Conclusion

This study suggests that dysbiosis of the gut microbiota in CI patients with enriched SCFAs producer, including *Odoribacter*, *Akkermansia*. This dysbiosis was correlation with the outcomes and might be used as biomarkers for the long-term outcome which clearly deserves further study.

## Methods

### Subjects

Total of 30 CI patients were selected. They were recruited from Qilu Hospital (Jinan, China) between May 2017 and January 2018, and had been diagnosed by skull computed tomography examination. The subjects enrolled in this study received and written the informed consent. The National Institutes of Health Stroke Scale (NIHSS) > 4 was defined as severe stroke. In addition, 30 healthy volunteers who were examined to ensure that they had no metabolic, cardiovascular or cerebrovascular diseases or cancer were selected as the normal group. All these individuals did not receive antibiotics or probiotic before 4 weeks prior to the specimen collection. This study was approved by the Ethical Committee of the Qilu Hospital of Shandong University (Jinan, China).

### Sample collection and 16S rRNA sequencing

The first available fecal samples of CI patients after admission (within 48 h) were collected. The samples and its genomic DNA were immediately frozen in liquid nitrogen and then stored at − 80 °C. Genomic DNA of microbes in fecal samples was extracted by CTAB assay. About 1 mg of each sample was used for DNA quantification by a NanoDrop ND-1000 spectrophotometer (NanoDrop Technologies, Wilmington, DE, USA).

For analyzing the microbial populations, amplification of the V1-V2 region of the 16S rRNA gene was performed via barcode-indexed primers 27F: 5′ AGAGTTTGATCMTGGCTC G, 338R-I 5′ GCWGCCTCCCGTAGGAGT, and 338R-II 5′ GCWGCCACCCGTAGGTGT). Then, we used the QIAquick PCR Purification Kit (Qiagen, Barcelona, Spain) to purified PCR amplicons and the products were quantified by a spectrophotometer as before. After that, it was pooled in equal concentration and 2 nM of pooled amplicons were then sequenced by Illumina MiSeq sequencer following the standard protocols.

### Analysis of the microbiota

Trimmomatic was used to demultiplexed and quality-filtered the Raw data. The obtained data was merged by FLASH compliance with rules: (i) The reads attaining an average quality score less than 20 in a sliding window of 50 bp were deleted. (ii) Primers were only allowing 2 nucleotides mismatching, meanwhile reads containing ambiguous bases were deleted. (iii) Sequences containing overlap over 10 bp were merged through the overlap sequence.

Majorbio I-Sanger Cloud Platform was used to analyze the microbiota (https://www.i-sanger.com). We used the UPARSE to clustered the operational taxonomic units (OTUs) with a cutoff of 97% similarity (version 7.1) as well as UCHIME were used to identified and removed chimeric sequences. RDP Classifier was used to analyzed the taxonomy of each sequence through algorithm against the Silva (SSU128) 16S rRNA database by a confidence threshold of 70% (http://rdp.cme.msu.edu/).

We normalized the OTUs abundance of each sample based on the least sequences number of all samples and removed the rare OTUs. Then, it was used to analyze the alpha diversity and beta diversity by QIIME tool. Meanwhile, linear discriminant analysis (LDA) effect size (LEfSe) analyses were carried out through the LEfSe tool with an LDA of 2.0. The analysis of similarity (ANOSIM) test were used to evaluate the differences of microbial structure between CI patients and healthy control.

### Biochemical assays

Blood samples were gathered from patients with stroke at admission and from healthy subjects at physical examination center. Serum levels of low-density lipoprotein (LDL), glucose of blood (GLU), high-density lipoprotein (HDL), uric acid (UA), triglycerides (TG), and homocysteine (HCY) were measured using standard techniques.

### Statistic methods

All data are demonstrated as mean ± SD. The normality of the distribution had been demonstrated with Kolmogorov-Smirnoff statistic of the data. The Chi-square-tests was used to assess gender. The T-tests was used for analysis of continuous variables. Spearman’s rank correlation was used to analysis of statistical dependence of continuous variables. *P*-values < 0.05 were considered as statistically significant. Analyses were carried out through the SPSS statistical package, version 24.0.

## Additional files

Additional file 1: **Table S1.** The serum index of CI patient and healthy control. (PDF 140 kb)

Additional file 2: **Table S2.** The severity and outcome of CI patients. (PDF 107 kb)

## Data Availability

All data generated or analyzed during this study are included in this published article.

## Abbreviations

CI: Cerebral ischemic stroke
GLU: Glucose of blood
HCY: Homocysteine
HDL: High-density lipoprotein
LDL: Low-density lipoprotein
OTU: Operational taxonomic unit
TG: Triglycerides
UA: Uric acid
