# Supplementary material for: Change of intestinal microbiota in cerebral ischemic stroke patients
Source: BMC Microbiol. 2019 Aug 19;19:191. doi: 10.1186/s12866-019-1552-1 (PMC6700817; doi:10.1186/s12866-019-1552-1)
Supplement: Supplementary file 1 — Table S1. The serum index of CI patient and healthy control. (PDF 140 kb) [file 12866_2019_1552_MOESM1_ESM.pdf]

Additional file 1: Table S1 The serum index of CI patients and healthy control.

Sample	TG	LDL	HDL	UA	Glu	Hcy
CI1	1.65	1.32	1.39	316	5.34	11.4
CI12	0.83	0.76	0.92	211	5.9	9.1
CE14	1.32	1.64	1.03	292	4.63	22.3
CE15	1.35	1.86	1.22	288	4.85	10.5
CE2	1.52	3.05	1.23	308	4.9	18.2
CE22	1.25	2.61	0.93	429	5.22	8.9
CE23	0.99	1.63	1.17	282	5.2	12.3
CE24	1.45	2.00	1.15	265	4.2	10.3
CE25	1.13	1.81	0.84	192	6.1	17.6
CE28	1.2	2.41	1.2	222	4.28	12
CE30	8.4	2.01	1.2	420	6.5	6.8
CE32	0.59	1.26	1.36	305	4.94	13.6
CE37	1.2	2.96	1.28	376	3.88	21.3
CE38	1.8	1.16	0.57	264	5.36	10.2
CE4	0.84	2.94	1.28	265	4.05	8.2
CE40	1.7	1.78	1.03	332	5.6	13.2
CE41	1.61	2.01	1.19	373	4.9	15.2
CE43	1.1	1.94	1.13	265	4.18	11.4
CE44	0.59	2.25	1.2	258	5.27	7.8
CE45	0.84	2.53	1.37	305	4.45	16.3
CE46	3.01	2.05	0.97	285	4.88	10.3
CE48	1.04	2.02	0.96	295	5.96	19.5
CE49	1.19	1.74	0.87	295	4.39	9.7
CE5	1.35	2.61	0.83	316	4.54	10.6
CE50	0.65	2.19	1.08	208	5.13	13.9
CE53	0.83	2.85	1.29	285	4.03	14.4
CE56	1.09	3.29	1.12	295	5.26	12.6
CE58	1.66	3.11	1.28	475	4.27	14.8
CE6	1.29	3.64	1.22	362	5.15	14.6
CE8	1.24	2.68	0.96	330	4.19	11.1
H10	1.71	2.08	0.83	345	5.22	12.4
H11	1.38	2.57	1.21	268	5.28	14.9
H13	1.03	1.77	1.35	161	5.58	12
H16	1.55	1.86	1.07	223	5.8	12.3
H17	1.25	2.82	0.97	368	6.9	19.7
H18	0.96	2.08	1.22	299	4.49	11.6
H19	1.32	1.48	1.35	255	4.69	6.7
H20	1.6	2.91	1.19	391	7.22	11.1
H21	0.86	2.39	1.75	236	5.38	9.7
H26	1.5	2.15	1.25	270	4.8	8.5
H27	1.61	2.29	1.07	228	4.87	10.2
H29	0.91	3.14	1.51	253	6.5	8.9

H3	0.94	1.46	1.03	251	4.03	18.2
H31	1.35	2.02	1.5	302	4.9	8.8
H33	1.25	2.25	1.05	345	5.2	12.8
H34	1.31	2.63	1.25	273	4.38	10.5
H35	1.78	1.25	0.94	273	4.38	18.7
H36	1.53	2.2	1.5	355	4.95	10.2
H39	1.66	2.62	1.36	320	4.95	12.2
H42	1.2	2.31	1.36	294	5.52	10.6
H47	1.7	3.16	1.33	380	5.71	10.4
H51	0.65	2.19	1.08	208	5.13	13.9
H52	0.8	1.72	1.14	248	5.51	11.2
H54	1.58	2.05	1.55	265	4.8	12.2
H55	1.13	2.96	1.71	246	4.03	14.4
H57	1.08	2.21	1.37	268	5.65	11.6
H59	1.35	2.97	1.29	155	5.88	14.1
H60	1.52	2.25	1.52	286	4.95	10.8
H7	1.59	2.39	1.75	284	4.23	20.8
H9	1.74	2.99	1.11	419	4.99	11.5

LDL, low-density lipoprotein; HDL high-density lipoprotein, GLU, glucose of blood; UA, uric acid; TG, triglycerides; HCY, homocysteine
