# Supplementary material for: Change of intestinal microbiota in cerebral ischemic stroke patients
Source: BMC Microbiol. 2019 Aug 19;19:191. doi: 10.1186/s12866-019-1552-1 (PMC6700817; doi:10.1186/s12866-019-1552-1)
Supplement: Supplementary file 2 — Table S2. The severity and outcome of CI patients. (PDF 107 kb) [file 12866_2019_1552_MOESM2_ESM.pdf]

Additional file 2: Table S2 The severity and outcome of CI patients

No.	NIHSS 1W	NIHSS 1M	mRS
CI15	11	8	5
CI24	11	8	5
CI32	11	9	5
CI1	9	6	4
CI4	8	3	2
CI48	6	5	3
CI 2	5	4	3
CI38	5	2	1
CI43	5	4	3
CI45	5	4	3
CI56	5	3	2
CI30	4	2	1
CI50	4	3	2
CI25	3	2	1
CI40	3	2	1
CI44	3	1	1
CI49	3	2	1
CI5	2	2	1
CI6	2	2	1
CI12	2	1	1
CI41	2	2	1
CI46	2	2	1
CI53	2	2	1
CI58	2	1	1
CI8	1	1	1
CI14	1	1	1
CI22	1	1	0
CI23	1	1	1
CI28	1	0	0
CI37	1	1	0

NIHSS, National Institutes of Health Stroke Scale
mRS, modulate RANK score
